# Phylogenetic Characterization Reveals Prevalent Shigella flexneri ST100 Clone in Beijing, China, 2005 to 2018

**DOI:** 10.1128/mSphere.00161-20

**Published:** 2020-07-15

**Authors:** Lang Yang, Bing Lü, Quanyi Wang, Kaiying Wang, Yanfeng Lin, Chaojie Yang, Shaofu Qiu, Peng Li, Hongbin Song

**Affiliations:** a Academy of Military Medical Sciences, Beijing, China; b Chinese PLA Center for Disease Control and Prevention, Beijing, China; c Institute for Infectious Disease and Endemic Disease Control, Beijing Center for Disease Prevention and Control, Beijing Research Center for Preventive Medicine, Beijing Key Laboratory of Diagnostic and Traceability Technologies for Food Poisoning, Beijing, China; Escola Paulista de Medicina/Universidade Federal de São Paulo

**Keywords:** *S. flexneri*, whole-genome sequencing, MDR determinants, population dynamics, clonal evolution

## Abstract

Shigella flexneri is a major cause of bacillary dysentery in Beijing, China. The genetic features and population structure of locally circulating clones remained unclear. In this study, we sequenced the genomes of 93 S. flexneri isolates from patients in Beijing from 2005 to 2018. Phylogenetic analysis revealed a predominant lineage comprised of ST100 isolates that had acquired an extensive repertoire of antimicrobial resistance determinants. A rapid local expansion of the largest clade of this lineage began in 2008 and gradually resulted in the dominance of serotype 2a. Other clades showed substantial evidence of interregional spread from other areas of China. Another lineage consisting of ST18 isolates was also identified and appeared to have persisted locally for nearly 6 decades. These findings suggest that S. flexneri epidemics in Beijing were caused by both local expansion and interregional transmission.

**IMPORTANCE** Beijing is the largest transportation hub in China, with a highly mobile population. Shigella flexneri is a major cause of bacillary dysentery in Beijing. However, little is known about the genetic features and population structure of locally circulating S. flexneri clones. Whole-genome sequencing of 93 S. flexneri isolates revealed that S. flexneri epidemics in Beijing were predominantly caused by an ST100 clone. Interregional spread, rapid local expansion, and acquirement of antimicrobial resistance determinants have cocontributed to the epidemics of this clone. Another ST18 clone was also identified and showed long-term colonization in Beijing. Our study provides comprehensive insights into the population structure and evolutionary history of S. flexneri in Beijing.

## Abstract (precis)

Beijing is the largest transportation hub in China, with a highly mobile population. Shigella flexneri is a major cause of bacillary dysentery in Beijing. However, little is known about the genetic features and population structure of locally circulating S. flexneri clones. Whole-genome sequencing of 93 S. flexneri isolates revealed that S. flexneri epidemics in Beijing were predominantly caused by an ST100 clone. Interregional spread, rapid local expansion, and acquirement of antimicrobial resistance determinants have cocontributed to the epidemics of this clone. Another ST18 clone was also identified and showed long-term colonization in Beijing. Our study provides comprehensive insights into the population structure and evolutionary history of S. flexneri in Beijing.

## INTRODUCTION

Shigellosis, an acute bacterial enteritis caused by *Shigella* spp., imposes major public health burdens worldwide. There are approximately 164.7 million cases of *Shigella* infections globally per year, resulting in 1.1 million deaths (1). The majority of deaths occur in children <5 years of age (1, 2). In China, *Shigella* spp. account for 0.8 to 1.7 million episodes of bacillary dysentery and up to 200,000 hospitalizations annually (3).

The genus *Shigella* is classified into four subgroups, S. flexneri, S. sonnei, S. boydii, and S. dysenteriae, based on biochemical and serological profiles. S. flexneri is the predominant species found in developing countries (1) such as China (3). Analysis of S. flexneri isolates collected worldwide reveals seven phylogenetic groups with distinct geographic ranges and abilities to colonize and persist in local areas over extended time periods (4). Horizontal transfer of a single azithromycin resistance plasmid facilitated the epidemic emergence of S. flexneri 2a and 3a (5). Genomic analysis of Chinese S. flexneri isolates also indicated that multidrug resistance was the crucial evolutionary adaptation driving the epidemic spread of an S. flexneri ST91 (later renamed ST100) clone in Henan, Shanxi, Anhui, and Gansu provinces (6). Beijing, the capital of China, is a developed and populous metropolis with 21.7 million people. The city serves as the largest domestic transportation hub of a highly mobile population. Although S. flexneri is a major cause of bacillary dysentery in Beijing (7), little is known about the molecular characteristics and population structure of locally circulating isolates.

In this study, we performed whole-genome sequencing (WGS) of 93 S. flexneri isolates from Beijing. Our analysis revealed that the S. flexneri population in Beijing features a dominant ST100 lineage that has acquired multiple antimicrobial resistance (AMR) genetic elements. Both local persistence and interregional transmission contributed to S. flexneri epidemics in Beijing.

## RESULTS

### Bacterial isolates and distribution of serotypes.

Ninety-three S. flexneri isolates were collected from 2005 to 2018. The isolates were grouped into six S. flexneri serotypes consisting of 1a, 2a, 2b, X, Xv, and Yv. Serotypes 2a and Xv were the predominant serotypes, accounting for 52.7% and 26.9% of all isolates, respectively. Serotype Xv was the most frequently identified serotype among isolates collected before 2011 (13/17, 76.5%), while 2a dominated after 2012 (48/76, 63.2%).

### Predominance of an ST100 S. flexneri lineage.

**FIG 1:**
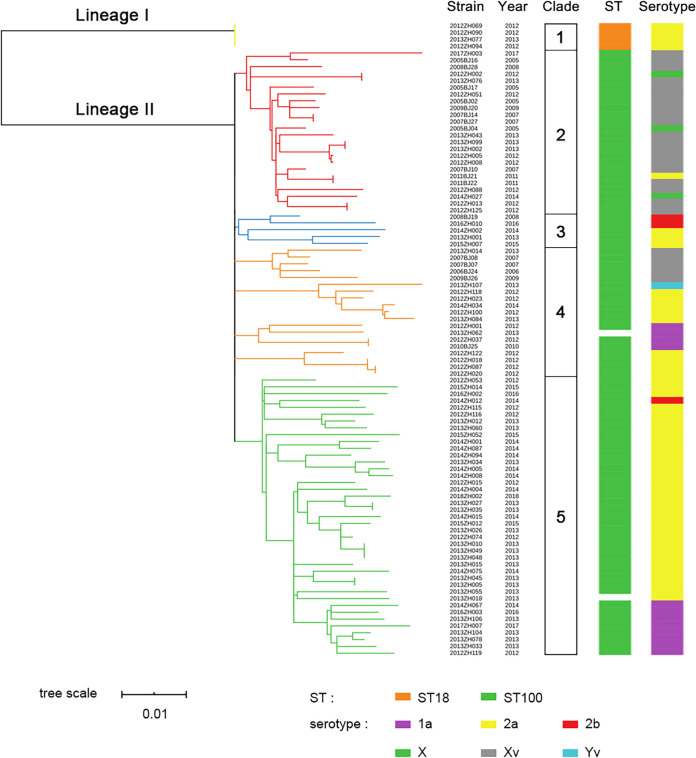
Phylogenetic tree of S. flexneri isolates from Beijing and correlation with sequence types and serotypes. Golden, red, blue, orange, and green branches indicate clades 1 to 5, respectively. Sequence types and serotypes are shown with colored bars.

The genetic structure of the S. flexneri bacterial population was determined by a phylogenetic analysis based on WGS. A total of 3,303 chromosomal single nucleotide polymorphisms (SNPs) in the core genome, excluding those in repeated and phage regions, were identified in the 93 genomes. A maximum-likelihood (ML) phylogenetic tree was constructed and demonstrated two distinct lineages and five clades (Fig. 1).

Lineage I corresponded to clade 1, which was composed exclusively of 4 isolates of serotype 2a belonging to sequence type 18 (ST18). Three of the isolates were recovered in 2012, and one in 2013. Sequence alignment revealed that the average nucleotide identity among the 4 isolates ranged from 99.86% to 99.98%, indicating extremely close relatedness.

Lineage II differed from lineage I by 701 SNP loci. Lineage II consisted of 87 isolates belonging to ST100 and 2 isolates (2013ZH062 and 2013ZH018) with undetermined sequence types. A point mutation at position 390 (C to T) was revealed in the housekeeping gene allele *lysP23* of ST100 relative to *lysP11* of ST18. Lineage II showed a high degree of genetic diversity and was subdivided into clades 2, 3, 4, and 5. The node of clade 2 was supported by 1,236 SNPs. Clade 2 consisted of 20 isolates of Xv, 3 of X, and 1 of 2a, of which 9 isolates were collected before 2009. Isolates of X and 2a were dispersed in this clade, indicating several independent serotype conversions.

Clade 3 contained 3 isolates of serotype 2a and 2 isolates of 2b and exhibited 251 SNPs. Clade 4 was further divided into subclades 1 to 4. There were 200, 194, 103, and 201 SNPs marking the internal node for each subclade, respectively. Subclade 1 contained 5 isolates of serotype Xv, four of which were collected before 2009; subclade 2 consisted of 5 isolates of 2a and 1 of Yv; subclade 3 was comprised of 4 isolates of 1a; subclade 4 included 4 isolates of 2a. In a multilocus sequence typing (MLST) analysis of 15 genes, isolate 2013ZH062 in subclade 3 shared 14 identical housekeeping genes with ST100 but had a different allele of *mtlD40*, relative to the *mtlD15* allele of ST100. The mannitol-1-phosphate dehydrogenase encoded by *mtlD40* has lost a proline compared with that encoded by *mtlD15*, which might impact the mannitol catabolism of S. flexneri. The tryptophanase operon, including three adjacent genes, *tnaL*, *tnaA*, and *tnaB*, was absent in subclade 4, which might hamper the conversion of tryptophan to indole in S. flexneri (8).

Clade 5 was the largest clade in lineage II; contained 32 isolates of 2a, 8 of 1a, and 1 of 2b; and exhibited 546 SNPs. All isolates were collected after 2012. The 8 isolates of 1a were closely related and grouped together as a subclade. MLST analysis revealed that isolate 2013ZH018 of 2a differed from the ST100 isolates by a novel allele of *aroE*, which had one synonymous nucleotide mutation compared to *aroE10* of ST100.

### Beijing lineages in the global S. flexneri evolution.

**FIG 2:**
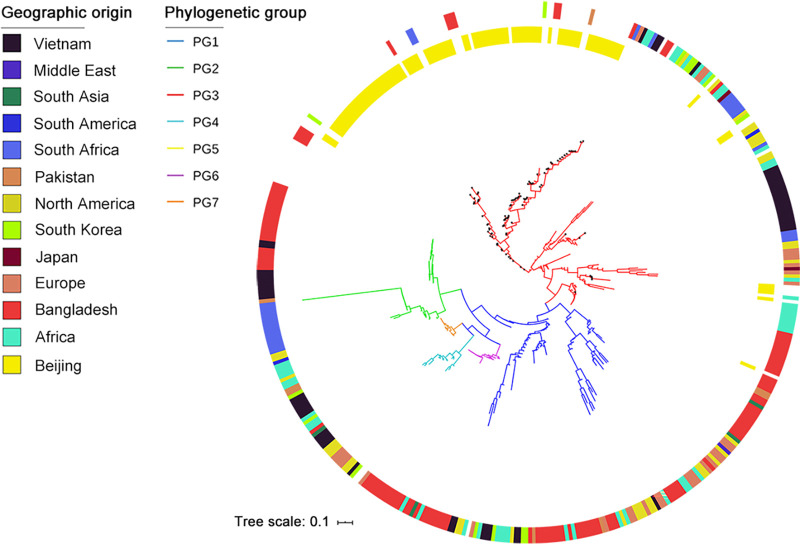
Phylogenetic tree of 93 S. flexneri isolates from Beijing and 348 global isolates. Phylogenetic groups are denoted by colored branches. Geographic origins of isolates are presented by surrounding colored blocks.

S. flexneri was subdivided into seven phylogenetic groups (PGs) (4). To reveal the role of the Beijing lineages in the S. flexneri evolution, a phylogenetic tree was constructed with 348 global and 93 Beijing S. flexneri isolates (Fig. 2). Tree topology revealed that Beijing lineages I and II clustered within PG3 but were classified into different branches. Beijing lineage I showed a close relationship to isolates from Africa, while Beijing lineage II was clustered with isolates from countries surrounding China, such as Bangladesh, South Korea, and Pakistan, suggesting that these two lineages have different evolutionary origins.

### Coexistence of local expansion and interregional transmission.

**FIG 3:**
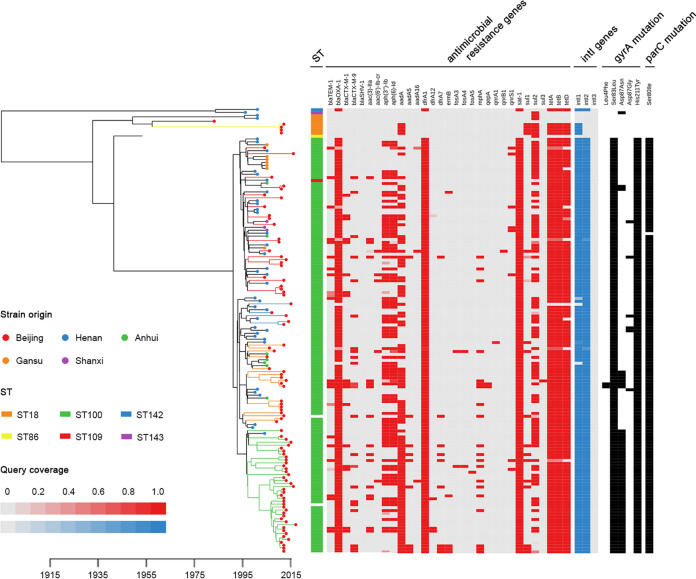
Bayesian maximum clade credibility phylogeny for 93 S. flexneri isolates from Beijing and 59 isolates from China. Golden, red, blue, orange, and green branches denote clades 1 to 5, respectively. Geographical origins of strains are indicated by colored circles. Sequence types are shown with colored bars. Red and blue indicate the query coverage against resistance genes and *intI* genes, respectively. Black bars denote the presence of point mutations in *gyrA* and *parC*.

To investigate the temporal and geographical history of S. flexneri in Beijing, the 93 S. flexneri genomes from Beijing and 59 previously published S. flexneri genomes from China (6) were evaluated in a Bayesian evolutionary analysis (Fig. 3). The mean substitution rate was estimated to be 2.2 × 10^−4^ per site per year among the 10,886 chromosomal SNP loci, corresponding to a genome-wide substitution rate of 5.2 × 10^−7^ per site per year. This substitution rate was consistent with previous estimates for S. flexneri (3.2 × 10^−7^ per site per year [6] and 6.46 × 10^−7^ to 9.54 × 10^−7^ per site per year [4]).

Tree topology revealed that all ST18 isolates in China were closely related. All 4 ST18 isolates of lineage I in this study fell into the same clade with a previous strain, 301, also isolated in Beijing in 1984 (9), and were distinct from other Chinese ST18 strains. This regionally restricted ST18 clone in Beijing could be traced back to a most recent common ancestor (MRCA) that arose in 1958.

Lineage II isolates also clustered with other ST100 isolates and formed the predominant group in China. The estimated time to the MRCA for the ST100 group is 26 years. Clades 2 to 4 from Beijing clustered with isolates from other Chinese locations, indicating cross-regional spread. Clade 2 isolates were geographically dispersed, with isolates from Henan, Anhui, Gansu, and Shanxi; clade 3 isolates were closely related to isolates from Henan; clade 4 isolates grouped with isolates from Henan and Anhui. Clades 3 and 4 evolved from an MRCA that emerged in 1995. The MCRA of clade 2 was distinct from those of clades 3 and 4 but was estimated to have also emerged circa 1995. In contrast, the Beijing clade 5 isolates were closely related and did not cluster with previous Chinese isolates, with the exception of a single isolate, 2005002 from Henan in 2005, suggesting independent evolution and local expansion. This clade evolved from a recent MRCA dating to 1998 and has rapidly expanded since 2008.

### Acquisition of antimicrobial resistance determinants.

Our study yielded substantial evidence of increased acquisition of genetic elements encoding drug resistance in Beijing S. flexneri isolates compared to previous Chinese isolates. Genomic analysis revealed that class 1 integron and tetracycline resistance genes (*tetA*, *tetB*, and *tetD*) were introduced into the Beijing lineage I in 2011. The class 1 integrons carried a cassette with only the *aadA1* gene. In contrast to lineage I, the Beijing lineage II isolates showed a greater accumulation of AMR determinants. All lineage II isolates harbored class 2 integrons carrying cassettes with *dfrA1*-*sat1*-*aadA1* and exhibited *gyrA* mutations of Ser83Leu and His211Tyr and the *parC* mutation of Ser80Ile. In addition, most of the lineage II isolates possessed another class 1 integron carrying an additional *bla*_OXA-1_ gene. Evolutionary analysis revealed that these AMR genetic elements were acquired at an early time near the emergence of the Chinese ST100 group circa 1992. Intriguingly, the *gyrA* mutation of Asp87Asn was introduced to the Beijing lineage II on three independent occasions. On one occasion it was maintained in the recently expanded clade 5. Further results revealed that the *bla*_CTX-M_, *aac(3)*-*IIa*, and *mphA* resistance genes were prevalent in Beijing lineage II but rarely detected in other Chinese ST100 isolates. These AMR genes were widely dispersed across the tree, indicating numerous independent acquisitions. Importantly, a novel *gyrA* gene point mutation of Leu4Phe was acquired by two Beijing isolates, 2012ZH100 and 2013ZH084, in 2011. The relevance of this mutation to phenotypic quinolone resistance requires further study.

## DISCUSSION

Our study demonstrated that S. flexneri epidemics in Beijing were caused predominantly by a single clone, ST100. A previous study also revealed the prevalence of the ST100 clone in Henan, Shanxi, Gansu, and Anhui provinces (6). As the largest domestic transportation hub, Beijing is the focal point of vast interregional travel and food importation. It was not surprising to find frequent interregional spread of the ST100 clone between Beijing and the above-named provinces. Our study found that a recently emerged ST100 sublineage expanded rapidly in the local region of Beijing circa 2008. The wide distribution of this sublineage since 2012 led 2a to replace Xv as the most prevalent serotype. These findings indicate that S. flexneri epidemics in Beijing were caused by both interregional transmission and local expansion. Although no direct epidemiological linkage was found, another ST18 clone showed close phylogenetic relationship to an early S. flexneri strain, 301, suggesting long-term local persistence of S. flexneri.

The overuse of antibiotics in China is alarming (10). The Beijing ST100 clone has evolved with an expanded repertoire of AMR genetic elements, indicating that antibiotic selection pressure may be the crucial evolutionary factor driving its prevalence. We observed an ongoing trend of accelerated AMR gene acquisition in Beijing S. flexneri isolates compared to isolates from other geographic locations. However, an epidemiological study demonstrated that antibiotic consumption in Beijing general hospitals is relatively low compared to that of hospitals in other regions (11). The causes of increased AMR still require further investigation. In addition, most of the newly acquired AMR determinants were introduced independently to the Beijing S. flexneri bacterial population and seemed to result in very limited expansions. This epidemiological feature differed remarkably from that of S. sonnei, among which acquisition of antimicrobial resistance genes drove clones’ rapid global dissemination (12). Previous studies have found that S. flexneri showed strong viability in water (13), implying that persistence in the environment might have enabled S. flexneri to be less affected by selection for antimicrobial resistance. Longitudinal studies need to be undertaken to reveal the potential effects of these AMR genetic elements on the local population structure.

In summary, WGS of 93 S. flexneri isolates demonstrated that S. flexneri epidemics in Beijing were predominantly caused by an ST100 clone with increasing AMR determinants. This clone was introduced through interregional spread from other areas of China and underwent rapid local expansion since 2008. Another ST18 clone was also identified and was found to have persisted in Beijing over an extended time period. Our study provides comprehensive insights into the population structure and evolutionary history of S. flexneri in Beijing.

## MATERIALS AND METHODS

### Bacterial strains and serotyping.

From 2005 to 2018, 93 S. flexneri isolates were collected through routine surveillance from patients contracting diarrhea in Beijing. Epidemiological data were recorded for each isolate (see Table S1 in the supplemental material). All S. flexneri strains were serotyped by slide agglutination using both commercially available monovalent antisera (Denka Serken, Japan) and monoclonal antibody reagents (Reagensia AB, Sweden) following the manufacturer’s instructions.

10.1128/mSphere.00161-20.1 TABLE S1 Details of S. flexneri isolates from Beijing with coverage of antimicrobial resistance determinants. Download Table S1, PDF file, 0.1 MB. Copyright © 2020 Yang et al. 2020 Yang et al. This content is distributed under the terms of the Creative Commons Attribution 4.0 International license.

### Whole-genome sequencing.

Genomic DNA was extracted from isolates using a High Pure PCR template preparation kit (Roche). The sequence library was prepared using a NEBNext DNA preparation kit (Illumina, Inc., San Diego, CA), and whole-genome sequencing (WGS) was performed using an Illumina MiSeq platform for 17 of the isolates in our lab. The remaining 76 isolates were sequenced using an Illumina NovaSeq 6000 system at Novogene Company (Beijing, China). Paired-end reads were assembled *de novo* using SPAdes (v3.6.2) (14).

### Phylogenetic and resistance analysis.

AMR genes were identified by aligning genome sequences against the Comprehensive Antibiotic Resistance Database (15). Sequence types were determined through a multilocus sequence typing (MLST) web server (16).

Paired-end reads were mapped onto the reference genome of S. flexneri serotype 2a strain 301 (accession number AE005674) using a Burrows-Wheeler Aligner (BWA) (v0.7.12) (17). Single nucleotide polymorphisms (SNPs) were identified using SAMtools (v1.3) (18). Repetitive sequences and phage regions were identified using the nucmer (MUMmer v3.0) (19) and phage search tool (PHAST) (20) programs, respectively, and excluded from the SNP analysis. The final chromosomal SNPs in the core genome were concatenated and aligned to construct a maximum-likelihood (ML) phylogenetic tree using RAxML (v8.2.4) (21) with a general time-reversible (GTR) model and a gamma distribution based on 1.000 bootstraps. The phylogenetic tree was visualized using iTOL (v4) (22).

To understand the evolutionary dynamics of S. flexneri, 59 previously published genomes of S. flexneri from China (6) were downloaded for a Bayesian Evolutionary Analysis by Sampling Trees (BEAST) analysis (23). SNPs were identified as described above. The BEAST analysis was performed using a GTR model with a gamma distribution; tip dates were defined as the year of isolation. A relaxed molecular clock (uncorrelated lognormal distribution) and a Bayesian skyline model were selected. Five independent runs were performed using chains of 100 million generations each with sampling every 1,000 generations. The estimates of the runs were combined using LogCombiner (v.1.8.4), excluding the first 10% of states in each chain as burn-in. The parameter and tree estimates were analyzed using Tracer (v1.8.4) and TreeAnnotator (v.1.8.4).

### Ethics statement.

This study was conducted in accordance with the requirements of the institutional ethics committee of the Academy of Military Medical Sciences. All the isolates were collected through routine surveillance, and informed consent was not required as no identifiable patient data were included in this study.

### Accession number(s).

The shotgun whole-genome sequences of 93 S. flexneri isolates have been deposited in NCBI under accession no. WPDI00000000 to WPGW00000000 (BioProject identification number PRJNA590276) (see Table S1 for details).
