# Supplementary material for: Phylogenetic Characterization Reveals Prevalent Shigella flexneri ST100 Clone in Beijing, China, 2005 to 2018
Source: mSphere. 2020 Jul 15;5(4):e00161-20. doi: 10.1128/mSphere.00161-20 (PMC7364208; doi:10.1128/mSphere.00161-20)
Supplement: TABLE S1 [file mSphere.00161-20-st001.pdf]

Strain	Accession number	Year of isolation	Origin	Serotype	ST	Lineage	Clade	Antimicrobial resistance genes																												
								*bla*TEM-1	*bla*OXA-1	*bla*CTX-M-1	*bla*CTX-M-9	*bla*SHV-1	*aac(3)-IIa*	*aac(6')-Ib-cr*	*aph(3'')-Ib*	*aph(6)-Id*	*aadA*	*aadA5*	*aadA16*	*dfrA1*	*dfrA12*	*dfrA7*	*ermB*	*fosA3*	*fosA4*	*fosA5*	*mphA*	*qepA*	*qnrA1*	*qnrB1*	*qnrS1*	*sat-1*	*sul1*	*sul2*	*sul3*	*tetA*
2005BJ02	WPGW00000000	2005	Beijing	Xv	100	II	5	1	0.3067	0	0	0	0	0	1	1	1	0	0	1	0	0	0	0	0	0	0	0	0	0	1	1	0	1	0	1
2005BJ04	WPGV00000000	2005	Beijing	X	100	II	5	0	1	0	0	0	0	0	1	1	1	0	0	1	0	0	0	0	0	0	0	0	0	0	0	1	0	1	0	1
2005BJ16	WPGU00000000	2005	Beijing	Xv	100	II	5	1	0.2828	0	0	0	0	0	0	0.7995	1	0	0.6139	1	0	0	0	0	0	0	0	0	0	0	1	1	0	1	0	0.5924
2005BJ17	WPGT00000000	2005	Beijing	Xv	100	II	5	0	1	1	0	0	0	0	1	1	1	0	0	1	0	0	0	0	0	0	0	0	0	0	0	1	0	1	0	1
2006BJ24	WPGS00000000	2006	Beijing	Xv	100	II	3	0	1	0	1	0	0	0	1	1	1	0	0	1	0	0	0	0	0	0	0	0	0	0	0	1	0	1	0	1
2007BJ07	WPGR00000000	2007	Beijing	Xv	100	II	3	0	1	0	0	0	0	0	1	1	1	0	0	1	0	0	0	0	0	0	0	0	0	0	0	1	0	1	0	1
2007BJ08	WPGQ00000000	2007	Beijing	Xv	100	II	3	0	0.3033	0	1	0	0	0	1	1	1	0	0	0.9368	0	0	0	0	0	0	0	0	0	0	0	1	0	1	0	0.71
2007BJ10	WPGP00000000	2007	Beijing	Xv	100	II	5	0	1	0	0	0	0.1288	1	1	1	1	0	1	1	0	0	0	0	0	0	0	0	0	1	0	1	1	1	0	1
2007BJ14	WPGO00000000	2007	Beijing	Xv	100	II	5	0	1	1	0	0	0	0	1	1	1	0	0	1	0.1543	0	0	0	0	0	0	0	0	0	0	1	0	1	0	1
2007BJ27	WPGN00000000	2007	Beijing	Xv	100	II	5	0	1	1	0	0	0	0	1	1	1	0	0	1	0	0	0	0	0	0	0	0	0	0	0	1	0	1	0	1
2008BJ19	WPGM00000000	2008	Beijing	2b	100	II	2	0	1	0	0	0	0	0	0	0	1	0	0	1	0	0	0	0	0	0	0	0	0	0	0	1	0	0.2644	0	1
2008BJ28	WPGL00000000	2008	Beijing	Xv	100	II	5	1	1	0	1	0	1	0	1	1	1	0	0	1	0	0	0	0	0	0	0	0	0	0	0	1	0	1	0	1
2009BJ20	WPGK00000000	2009	Beijing	Xv	100	II	5	0	1	0	0	0	0	0	1	1	1	0	0	1	0	0	0	0	0	0	0	0	0	0	0	1	0	1	0	1
2009BJ26	WPGJ00000000	2009	Beijing	Xv	100	II	3	0	1	0	1	0	0	1	0	0.7351	1	0	0	1	0	0	0	1	1	0	1	0	0	0	0	1	1	0	0	1
2010BJ25	WPGI00000000	2010	Beijing	1a	100	II	4	0	1	0	0	0	0	0	1	1	1	0	0	1	0	0	0	0	0	0	0	0	0	0	0	1	0	1	0	1
2011BJ21	WPGH00000000	2011	Beijing	2a	100	II	5	1	0.2896	1	0	0	1	0	0	0	1	0	0	0.9937	0	0	0	0	0	0	0	0	0	0	0	1	0	0	0	0.6471
2011BJ22	WPGG00000000	2011	Beijing	Xv	100	II	5	1	1	1	0	0	1	0	1	0.1337	1	0	0	1	0	0	0	0	0	0	0	0	0	0	0	1	0	0.5569	0	1
2012ZH001	WPGF00000000	2012	Beijing	1a	100	II	4	0	1	0	0	0	0	0	1	1	0.9861	0	0	1	0	0	0	0	0	0	0	0	0	0	0	1	0	1	0	1
2012ZH002	WPGE00000000	2012	Beijing	X	100	II	5	0	1	0	0	0	0	0	0	0	0.9861	0	0	1	0	0	0	0	0	0	0	0	0	0	0	1	0	0	0	1
2012ZH005	WPGD00000000	2012	Beijing	Xv	100	II	5	0	1	0	0	0	0	0	0	0	0.9861	0	0	1	0	0	0	0	0	0	0	0	0	0	0	1	0	0	0	1
2012ZH008	WPGC00000000	2012	Beijing	Xv	100	II	5	0	1	0	0	0	0	0	0	0	0.9861	0	0	1	0	0	0	0	0	0	0	0	0	0	0	1	0	0	0	1
2012ZH013	WPGB00000000	2012	Beijing	Xv	100	II	5	0	1	0	0	0	0	0	0.3391	0	0.9861	0	0	1	0	0	0	0	0	0	0	0	0	0	0	1	0	0	0	1
2012ZH015	WPGA00000000	2012	Beijing	2a	100	II	6	0	1	0	0	0	0	0	1	1	0.9861	0	0	1	0	0	0	0	0	0	0	0	0	0	0	1	0	1	0	1
2012ZH018	WPFZ00000000	2012	Beijing	2a	100	II	4	0.5302	1	1	0	0	0	0	0	0	0.9861	0	0	1	0	0	0	0	0	0	1	0	0	0	0	1	0	0	0	1
2012ZH020	WPFY00000000	2012	Beijing	2a	100	II	4	0	1	0	0	0	0	0	0	0	0.9861	0	0	1	0	0	0	0	0	0	0	0	0	0	0	1	0	0	0	1
2012ZH023	WPFX00000000	2012	Beijing	2a	100	II	3	0	1	0	0	0	0	0	1	1	0.9861	0	0	1	0	0	0	0	0	0	0	0	0	0	0	1	0	1	0	1
2012ZH037	WPFW00000000	2012	Beijing	1a	100	II	4	0	1	0	0	0	0	0	1	1	0.9861	0	0	1	0	0	0	0	0	0	0	0	0	0	0	1	0	1	0	1
2012ZH051	WPFV00000000	2012	Beijing	Xv	100	II	5	0	1	0	0	0	0	0	0	0	0.9861	0	0	1	0	0	0	0	0	0	0	0	0	0	0	1	0	0	0	1
2012ZH053	WPFU00000000	2012	Beijing	2a	100	II	6	0	1	0	0	0	0	0	1	1	0.9861	0	0	1	0	0	0	0	0	0	0	0	0	0	0	1	0	1	0	1
2012ZH069	WPFT00000000	2012	Beijing	2a	18	I	1	0	0	0	0	0	0	0	0	0	1	0	0	0	0	0	0	0	0	0	0	0	0	0	0	0	1	1	0	1
2012ZH074	WPFS00000000	2012	Beijing	2a	100	II	6	0	1	0	0	0	0	0	0	0	0.9861	0	0	1	0	0	0	0	0	0	0	0	0	0	0	1	0	0.0428	0	1
2012ZH087	WPFR00000000	2012	Beijing	2a	100	II	4	0	1	0	0	0	0	0	0	0	0.9861	0	0	1	0	0	0	0	0	0	0	0	0	0	0	1	0	0	0	1
2012ZH088	WPFQ00000000	2012	Beijing	Xv	100	II	5	1	1	0	1	0	1	0	1	1	0.9861	1	0	1	0	1	0	0	0	0	1	0	0	0	0	1	0.409	1	0	1
2012ZH090	WPFP00000000	2012	Beijing	2a	18	I	1	0	0	0	0	0	0	0	0	0	1	0	0	0	0	0	0	0	0	0	0	0	0	0	0	0	1	1	0	1
2012ZH094	WPFO00000000	2012	Beijing	2a	18	I	1	0	0	0	0	0	0	0	0	0	1	0	0	0	0	0	0	0	0	0	0	0	0	0	0	0	1	1	0	1
2012ZH100	WPFN00000000	2012	Beijing	2a	100	II	3	1	1	1	0.8141	0	1	0	1	1	0.9861	0	0	1	0	0	0	0	0	0	1	1	0	0	0	1	0	1	0	1
2012ZH115	WPFM00000000	2012	Beijing	2a	100	II	6	1	1	0	0	0	0	0	1	1	0.9861	0	0	1	0	0	0	0	0	0	0	0	0	0	0	1	0	1	0	1
2012ZH116	WPFL00000000	2012	Beijing	2a	100	II	6	0	1	0	0	0	0	0	0	0	0.9861	0	0	1	0	0	0	0	0	0	0	0	0	0	0	1	0	0	0	1
2012ZH118	WPFK00000000	2012	Beijing	2a	100	II	3	0	1	0	0	0	0	0	1	1	0.9861	0	0	1	0	0	0	0	0	0	0	0	0	0	0	1	0	1	0	1
2012ZH119	WPFJ00000000	2012	Beijing	1a	100	II	6	1	1	0	0	0	1	0	0	0	0.9861	0	0	1	0	0	0.9748	0	0	0	1	0	0	0	0	1	0	0	0	1
2012ZH122	WPFI00000000	2012	Beijing	2a	100	II	4	0	1	0	0	0	0	0	1	1	0.9861	0	0	1	0	0	0	0	0	0	0	0	0	0	0	1	0	1	0	1
2012ZH125	WPFH00000000	2012	Beijing	Xv	100	II	5	0	1	0	0	0	0	0	0	0	0.9861	0	0	1	0	0	0	0	0	0	0	0	0	0	0	1	0	0	0	1
2013ZH001	WPFG00000000	2013	Beijing	2a	100	II	2	0	1	0	0	0	0	0	0	0	0.9861	0	0	1	0	0	0	0	0	0	0	0	0	0	0	1	0	0	0	1
2013ZH002	WPFF00000000	2013	Beijing	Xv	100	II	5	0.5302	1	1	0	0	0	0	0.318	0	0.9861	0	0	1	0	0	0	0	0	0	0	0	0	0	0	1	0	0	0	1
2013ZH005	WPFE00000000	2013	Beijing	2a	100	II	6	0	1	0	0	0	0	0	0.3106	0	0.9861	0	0	1	0	0	0	0	0	0	0	0	0	0	0	1	0	0	0	1
2013ZH010	WPFD00000000	2013	Beijing	2a	100	II	6	0	1	0	1	0	0	0	0.3963	0	0.9861	1	0	1	0	1	0.9748	0	0	0	1	0	0	0	0	1	1	0.5031	0	1
2013ZH012	WPFC00000000	2013	Beijing	2a	100	II	6	0	1	0	0	0	0	0	0	0	0.9861	0	0	1	0	0	0	0	0	0	0	0	0	0	0	1	0	0	0	1
2013ZH014	WPFB00000000	2013	Beijing	Xv	100	II	3	0	1	0	0	0	0	0	0	0	0.9861	0	0	1	0	0	0	0	0	0	0	0	0	0	0	1	0	0	0	1
2013ZH015	WPFA00000000	2013	Beijing	2a	100	II	6	0	1	0	0	0	0	0	0	0	0.9861	0	0	1	0	0	0	0	0	0	0	0	0	0	0	1	0	0	0	1
2013ZH018	WPEZ00000000	2013	Beijing	2a	NEW	II	6	0	1	0	1	0	0	0	1	1	0.9861	0	0	1	0	0	0	0	0	0	0	0	0	0	0	1	0	1	0	1
2013ZH026	WPEY00000000	2013	Beijing	2a	100	II	6	0	1	0	0	0	0	0	0	0	0.9861	0	0	1	0	0	0	0	0	0	0	0	0	0	0	1	0	0	0	1
2013ZH027	WPEX00000000	2013	Beijing	2a	100	II	6	1	1	1	0	0	1	0	1	1	0.9861	0	0	1	1	0	0	0	0	0	0	0	0	0	0	1	0	1	0	1
2013ZH033	WPEW00000000	2013	Beijing	1a	100	II	6	1	1	0	0	0	1	0	1	1	0.9861	0	0	1	1	0	0	0	0	0	0	0	0	0	0	1	0	1	0	1
2013ZH034	WPEV00000000	2013	Beijing	2a	100	II	6	0	1	1	0.8141	0	0	0	1	1	0.9861	0	0	1	0	0	0	1	1	0	1	0	0	0	0	1	0	1	0	1
2013ZH035	WPEU00000000	2013	Beijing	2a	100	II	6	1	1	1	0	0	1	0	1	1	0.9861	0	0	1	1	0	0	0	0	0	0	0	0	0	0	1	0	1	0	1
2013ZH043	WPET00000000	2013	Beijing	Xv	100	II	5	0	1	0	1	0	0	1	1	1	0.9861	0	0	1	0	0	0	0	0	0	1	0	0	0	0	1	0.2485	1	0	1
2013ZH045	WPES00000000	2013	Beijing	2a	100	II	6	0	1	0	0	0	0	0	0	0	0.9861	0	0	1	0	0	0	0	0	0	0	0	0	0	0	1	0	0	0	1
2013ZH048	WPER00000000	2013	Beijing	2a	100	II	6	0	1	0	1	0	0	0	0	0	0.9861	1	0	1	0	1	0.9748	0	0	0	1	0	0	0	0	1	1	0	0	1
2013ZH049	WPEQ00000000	2013	Beijing	2a	100	II	6	0	1	0	1	0	0	0	0	0	0.9861	1	0	1	0	1	0.9748	0	0	0	1	0	0	0	0	1	1	0	0	1
2013ZH055	WPEP00000000	2013	Beijing	2a	100	II	6	0	1	0	1	0	0	0	1	1	0.9861	0	0	1	0	0	0	0	0	0	0	0	0	0	0	1	0	1	0	1
2013ZH060	WPEO00000000	2013	Beijing	2a	100	II	6	0	1	0	1	0	1	0	0	0	0.9861	1	0	1	0	1	0	0	0	0	1	0	0	0	0	1	1	0	0	1
2013ZH062	WPEN00000000	2013	Beijing	1a	NEW	II	4	0	1	0	1	0	1	0	0	0	0.9861	1	0	1	0	1	0	0	0	0	1	0	0	0	0	1	1	0.1995	0	1
2013ZH076	WPEM00000000	2013	Beijing	Xv	100	II	5	0	1	0	0	0	0	0	0	0	0.9861	0	0	1	0	0	0	0	0	0	0	0	0	0	0	1	0	0	0	1
2013ZH077	WPEL00000000	2013	Beijing	2a	18	I	1	0	0	0	0	0	0	0	0	0	1	0	0	0	0	0	0	0	0	0	0	0	0	0	0	0	1	1	0	1
2013ZH078	WPEK00000000	2013	Beijing	1a	100	II	6	0	1	0	0	0	0	0	0	0	0.9861	0	0	1	0	0	0	0	0	0	0	0	0	0	0	1	0	0	0	1
2013ZH084	WPEJ00000000	2013	Beijing	2a	100	II	3	1	1	1	0.8141	0	1	0	0	0	0.9861	0	0	1	0	0	0	0	0	0	1	1	0	0	0	1	0	0	0	1
2013ZH099	WPEI00000000	2013	Beijing	Xv	100	II	5	0.5302	1	1	0	0	0	0	0	0	0.9861	0	0	1	0	0	0	0	0	0	0	0	0	0	0	1	0	0	0	1
2013ZH104	WPEH00000000	2013	Beijing	1a	100	II	6	0	1	0	0	0	0	0	0	0	0.9861	0	0	1	0	0	0	0	0	0	0	0	0	0	0	1	0	0	0	1
2013ZH106	WPEG00000000	2013	Beijing	1a	100	II	6	0	1	0	0	0	0	0	1	1	0.9861	0	0	1	0	0	0	0	0	0	0	0	0	0	0	1	0	1	0	1
2013ZH107	WPEF00000000	2013	Beijing	Yv	100	II	3	0	1	0	0	0	0	0	1	1	0.9861	0	0	1	0	0	0	0	0	0	0	0	0	0	0	1	0	1	0	1
2014ZH001	WPEE00000000	2014	Beijing	2a	100	II	6	1	1	0	0	0	1	0	1	1	0.9861	1	0	1	0	1	0	0	0	0	1	0	0	0	1	1	1	1	0	0.4656
2014ZH002	WPED00000000	2014	Beijing	2a	100	II	2	0	1	0	1	0	0	0	1	1	0.9861	0	0	1	0	0	0	0	0	0	0	0	0	0	0	1	0	1	0	1
2014ZH004	WPEC00000000	2014	Beijing	2a	100	II	6	0	1	0	0	0	0	0	0	0	0.9861	0	0	1	0	0	0	0	0	0	0	0	0	0	0	1	0	0	0	1
2014ZH005	WPEB00000000	2014	Beijing	2a	100	II	6	0	1	0	0	0	0	0	0	0	0.9861	0	0	1	0	0	0	0	0	1	0	0	0	0	0	1	0	0	0	1
2014ZH008	WPEA00000000	2014	Beijing	2a	100	II	6	0.1972	1	1	0	0	0	0	1	1	0.9861	0	0	1	0	0	0	0	0	0	0	0	0	0	1	1	0	1	0	1
2014ZH012	WPDZ00000000	2014	Beijing	2b	100	II	6	0	1	0	0	0	0	0	1	1	0.9861	0	0	1	0	0	0	0	0	0	0	0	0	0	0	1	0	1	0	1
2014ZH015	WPDY00000000	2014	Beijing	2a	100	II	6	0	1	0	0	0	0	0	0	0	0.9861	0	0	1	0	0	0	0	0	0	0	0	0	0	0	1	0	0	0	1
2014ZH027	WPDX00000000	2014	Beijing	X	100	II	5	0	1	0	0	0	0	0	0	0	0.9861	0	0	1	0	0	0	0	0	0	0	0	0	0	0	1	0	0	0	1
2014ZH034	WPDW00000000	2014	Beijing	2a	100	II	3	1	1	1	0	0	1	0	0.2099	0.8508	0.9861	0	0	1	0	0	0	0	0	0	1	0	0	0	0	1	0	0.0722	1	1
2014ZH067	WPDV00000000	2014	Beijing	1a	100	II	6	0	1	0	0	0	0	1	1	1	0.9861	0	1	1	0	0	0	0	0	0	0.9956	0	0	0	0	1	1	1	0	1
2014ZH075	WPDU00000000	2014	Beijing	2a	100	II	6	0	1	0	1	0	0	0	1	1	0.9861	0	0	1	0	0	0	0	0	0	1	0	0	0	0	1	0	1	0	1
2014ZH087	WPDT00000000	2014	Beijing	2a	100	II	6	0	1	0	0	0	0	0	0	0	0.9861	0	0	1	0	0	0	0	0	0	0	0	0	0	0	1	0	0	0	1
2014ZH094	WPDS00000000	2014	Beijing	2a	100	II	6	0	1	0	0	0	0	0	0	0	0.9861	0	0	1	0	0	0	0	0	0	0	0	0	0	0	1	0	0	0	1
2015ZH007	WPDR00000000	2015	Beijing	2a	100	II	2	0	1	0	0	0	0	0	1	1	0.9861	0	0	1	0	0	0	0	0	0	0	0	0	0	0	1	0	1	0	1
2015ZH012	WPDQ00000000	2015	Beijing	2a	100	II	6	0	1	0	0	0	0	0	0	0	0.9861	0	0	1	0	0	0	0	0	0	0	0	0	0	0	1	0	0	0	1
2015ZH014	WPDP00000000	2015	Beijing	2a	100	II	6	1	1	1	0	0	1	0	1	1	0.9861	1	0	1	0	1	0	0	0	0	1	0	0	0	0	1	1	1	0	1
2015ZH052	WPDO00000000	2015	Beijing	2a	100	II	6	0	1	0	0	0	0	0	1	1	0.9861	0	0	1	0	0	0	0	0	0	0	0	0	0	0	1	0	1	0	1
2016ZH002	WPDN00000000	2016	Beijing	2a	100	II	6	0	1	0	0	0	0	0	1	1	0.9861	0	0	1	0	0	0	0	0	0	0	0	0	0	0	1	0	1	0	1
2016ZH003	WPDM00000000	2016	Beijing	1a	100	II	6	0	1	0	0	0	0	0	1	1	0.9861	0	0	1	0	0	0	0	0	0	0	0	0	0	0	1	0	1	0	1
2016ZH010	WPDL00000000	2016	Beijing	2b	100	II	2	0	0	0	0	0	0	0	1	1	1	0	0	1	0	0	0	0	0	0	0	0	0	0	0	1	0	1	0	0
2017ZH003	WPDK00000000	2017	Beijing	Xv	100	II	5	0	1	0	0	0	0	0	0	0	0.9861	0	0	1	0	0	0	0	0	0	0	0	0	0	1	1	0	1	0	1
2017ZH007	WPDJ00000000	2017	Beijing	1a	100	II	6	0	1	0	1	0	1	0	0	0	0.9861	1	0	1	0	1	0	0	0	0	1	0	0	0	0	1	1	0	0	1
2018ZH002	WPDI00000000	2018	Beijing	2a	100	II	6	0	1	0	0	0	0	0	0	0	0.9861	0	0	1	0	0	0	0	0	0	0	0	0	0	0	1	0	0	0	1

		intI genes			*gyrA* mutations					*parC* mutation
tetB	***tetD***	***intI1***	***intI2***	***intI3***	**Leu4Phe**	**Ser83Leu**	**Asp87Asn**	**Asp87Gly**	**His211Tyr**	**Ser80Ile**
1	0	1	1	0	0	1	0	0	1	1
1	1	1	1	0	0	1	0	0	1	1
0.5929	0	1	0.9631	0	0	1	0	0	1	1
1	1	1	1	0	0	1	0	0	1	1
1	1	1	1	0	0	1	0	0	1	1
1	1	1	1	0	0	1	0	0	1	1
0.7106	0	1	0.8164	0	0	1	0	0	1	1
1	1	1	1	0	0	1	0	0	1	1
1	1	1	1	0	0	1	0	0	1	1
1	0.4904	1	1	0	0	1	0	0	1	1
1	1	1	1	0	0	1	0	0	1	1
1	1	1	1	0	0	1	0	0	1	1
1	1	1	1	0	0	1	0	0	1	1
1	1	1	1	0	0	1	0	0	1	1
1	1	1	1	0	0	1	0	0	1	1
0.6476	0	1	0.8226	0	0	1	0	0	1	1
1	1	1	1	0	0	1	0	0	1	1
1	1	1	1	0	0	1	0	0	1	1
1	1	1	1	0	0	1	1	0	1	1
1	1	1	1	0	0	1	0	0	1	1
1	1	1	1	0	0	1	0	0	1	1
1	1	1	1	0	0	1	0	0	1	1
1	1	1	1	0	0	1	1	0	1	1
1	1	1	1	0	0	1	0	0	1	1
1	1	1	1	0	0	1	0	0	1	1
1	1	1	1	0	0	1	1	0	1	1
1	1	1	1	0	0	1	0	0	1	1
1	1	1	1	0	0	1	0	0	1	1
1	1	1	1	0	0	1	1	0	1	1
1	1	1	0	0	0	0	0	0	0	0
1	1	1	1	0	0	1	1	0	1	1
1	1	1	1	0	0	1	0	0	1	1
1	1	1	1	0	0	1	0	1	1	1
1	1	1	0	0	0	0	0	0	0	0
1	1	1	0	0	0	0	0	0	0	0
1	1	1	1	0	1	1	1	0	1	1
1	1	1	1	0	0	1	1	0	1	1
1	1	1	1	0	0	1	1	0	1	1
1	1	1	1	0	0	1	1	0	1	1
1	1	1	1	0	0	1	1	0	1	1
1	1	1	1	0	0	1	0	0	1	1
1	1	1	1	0	0	1	0	0	1	1
1	1	1	1	0	0	1	0	0	1	1
1	1	1	1	0	0	1	0	0	1	1
1	1	1	1	0	0	1	1	0	1	1
1	1	1	1	0	0	1	1	0	1	1
1	1	1	1	0	0	1	1	0	1	1
1	1	1	1	0	0	1	0	0	1	1
1	1	1	1	0	0	1	1	0	1	1
1	1	1	1	0	0	1	1	0	1	1
1	1	1	1	0	0	1	1	0	1	1
1	1	1	1	0	0	1	1	0	1	1
1	1	1	1	0	0	1	1	0	1	1
1	1	1	1	0	0	1	1	0	1	1
1	1	1	1	0	0	1	1	0	1	1
1	1	1	1	0	0	1	0	0	1	1
1	1	1	1	0	0	1	1	0	1	1
1	1	1	1	0	0	1	1	0	1	1
1	1	1	1	0	0	1	1	0	1	1
1	1	1	1	0	0	1	1	0	1	1
1	1	1	1	0	0	1	1	0	1	1
1	1	1	1	0	0	1	0	0	1	1
1	1	1	1	0	0	1	1	0	1	1
1	1	1	0	0	0	0	0	0	0	0
1	1	1	1	0	0	1	1	0	1	1
1	1	1	1	0	1	1	1	0	1	1
1	1	1	1	0	0	1	0	0	1	1
1	1	1	1	0	0	1	1	0	1	1
1	1	1	1	0	0	1	1	0	1	1
1	1	1	1	0	0	1	1	0	1	1
0.466	0	1	1	0	0	1	1	0	1	1
1	1	1	1	0	0	1	0	1	1	1
1	1	1	1	0	0	1	1	0	1	1
1	1	1	1	0	0	1	1	0	1	1
1	1	1	1	0	0	1	1	0	1	1
1	1	1	1	0	0	1	1	0	1	1
1	1	1	1	0	0	1	1	0	1	1
1	1	1	1	0	0	1	0	0	1	1
1	1	1	1	0	0	1	1	0	1	1
1	1	1	1	0	0	1	1	0	1	1
1	1	1	1	0	0	1	1	0	1	1
1	1	1	1	0	0	1	1	0	1	1
1	1	1	1	0	0	1	1	0	1	1
1	1	1	1	0	0	1	0	0	1	1
1	1	1	1	0	0	1	1	0	1	1
1	1	1	1	0	0	1	1	0	1	1
1	1	1	1	0	0	1	1	0	1	1
1	1	1	1	0	0	1	1	0	1	1
1	1	1	1	0	0	1	1	0	1	1
0	0	0	1	0	0	1	0	0	1	1
1	1	1	1	0	0	1	0	0	1	1
1	1	1	1	0	0	1	1	0	1	1
1	1	1	1	0	0	1	1	0	1	1
